# Mechanisms that allow vaccination against an oncolytic vesicular stomatitis virus-encoded transgene to enhance safety without abrogating oncolysis

**DOI:** 10.1038/s41598-021-94483-z

**Published:** 2021-07-27

**Authors:** Amanda W. K. AuYeung, Robert C. Mould, Ashley A. Stegelmeier, Jacob P. van Vloten, Khalil Karimi, J. Paul Woods, James J. Petrik, Geoffrey A. Wood, Byram W. Bridle

**Affiliations:** 1 Department of Pathobiology, Ontario Veterinary College, University of Guelph, Guelph, ON N1G 2W1 Canada; 2 Department of Clinical Studies, Ontario Veterinary College, University of Guelph, Guelph, ON N1G 2W1 Canada; 3 Department of Biomedical Sciences, Ontario Veterinary College, University of Guelph, Guelph, ON N1G 2W1 Canada; 4 Department of Pathobiology, Ontario Veterinary College, University of Guelph, Rm. 4834, Bldg. 89, 50 Stone Rd. E., Guelph, ON N1G 2W1 Canada

**Keywords:** Cancer, Cancer therapy, Cancer immunotherapy

## Abstract

Vaccination can prevent viral infections via virus-specific T cells, among other mechanisms. A goal of oncolytic virotherapy is replication of oncolytic viruses (OVs) in tumors, so pre-existing T cell immunity against an OV-encoded transgene would seem counterproductive. We developed a treatment for melanomas by pre-vaccinating against an oncolytic vesicular stomatitis virus (VSV)-encoded tumor antigen. Surprisingly, when the VSV-vectored booster vaccine was administered at the peak of the primary effector T cell response, oncolysis was not abrogated. We sought to determine how oncolysis was retained during a robust T cell response against the VSV-encoded transgene product. A murine melanoma model was used to identify two mechanisms that enable this phenomenon. First, tumor-infiltrating T cells had reduced cytopathic potential due to immunosuppression. Second, virus-induced lymphopenia acutely removed virus-specific T cells from tumors. These mechanisms provide a window of opportunity for replication of oncolytic VSV and rationale for a paradigm change in oncolytic virotherapy, whereby immune responses could be intentionally induced against a VSV-encoded melanoma-associated antigen to improve safety without abrogating oncolysis.

## Introduction

Oncolytic virotherapy represents a promising treatment modality within the field of cancer immunotherapy. Oncolytic viruses (OVs) preferentially replicate in cancer cells, often due to inherent defects in anti-viral signaling pathways as a result of the transformation process^1^. This allows OVs to mediate efficient antigen-presentation to T cells by providing target antigens from killed cancer cells and indirect co-stimulation by providing or inducing pathogen- and damage-associated molecular patterns. Unfortunately, immune responses induced by the in situ vaccination effect of OVs are dominated by responses to highly immunogenic viral backbone proteins, rather than self-derived tumor-associated antigens (TAAs) with low immunogenicity due to mechanisms governing central and peripheral tolerance. For the same reason, encoding TAAs into the genome of recombinant OVs fails to facilitate induction of substantial numbers of tumor-specific T cells when these viruses are used as primary vaccines^2^. Therefore, we developed a strategy to enhance the potential of oncolytic vesicular stomatitis virus (VSV) as a cancer vaccine. The VSV-vectored vaccine was not effective at priming T cell responses because the highly immunogenic backbone proteins of the replicating virus elicited a T cell response predominantly against the viral backbone, with a comparatively weak response to the encoded TAA^2^. However, replication-deficient viruses, such as an E1/E3-deleted human serotype 5 adenovirus, can be used to express a TAA under the control of a strong promoter. This causes the TAA to be preferentially expressed, leading to induction of relatively high-magnitude primary TAA-specific T cell responses^2^. These responses could be rapidly and dramatically boosted using attenuated oncolytic rhabdoviruses as secondary vaccines^3^. This translated into a substantial extension of survival time with an approximate 20% cure rate in a model that was otherwise incurable. The intention of this prime-boost method was to gain dual benefits: enhanced TAA-specific T cell responses, and acute debulking of tumors by VSV-mediated oncolysis. Indeed, in addition to induction of massive tumor-specific T cell responses, the VSV-vectored vaccine could replicate inside tumors^2^. Notably, boosting of T cell numbers could be accomplished at and even prior to the peak of a primary immune response^2,4^. This was traditionally thought to be the worst time to administer booster vaccines due to negative feedback regulation mediated by effector T cells killing antigen-presenting cells displaying cognate epitopes. However, this negative feedback regulation could be bypassed with VSV by virtue of its ability to promote antigen presentation in an immunologically privileged site in splenic B cell follicles, where memory T cells reside in proximity to antigen-presenting cells but in relative isolation from effector T cells^3^. Originally shown to work with VSV^2^, this method can also be used with the related rhabdovirus, Maraba virus^4,5^. Further, the method has been expanded from its initial pre-clinical application against melanomas, to target other tumor types including human papilloma virus-associated and prostate cancers^5–7^. Most importantly, the use of a melanoma-associated antigen 3 (MAGE-A3)-expressing Maraba virus as an oncolytic booster vaccine has entered four phase 1 and 2 human clinical trials to treat patients with advanced and/or metastatic MAGE-A3-expressing solid tumours, including melanomas, and in combination with a programmed cell death-1 receptor-specific antibody (i.e*.* pembrolizumab) against non-small cell lung cancers (ClinicalTrials.gov Identifiers: NCT02285816, NCT03618953, NCT02879760 and NCT03773744)^8^.

Retrospective analysis of pre-clinical data suggested that using VSV as a booster vaccine could make the virus safer. Specifically, hind-limb paralysis, a toxicity that can occur in mice treated with very high doses of neurotropic rhabdoviruses like VSV^9^, was not observed in mice that received primary heterologous vaccines against the virus-encoded TAA^2^. Presumably, this was because transgene-specific T cells could kill virus-infected cells, thereby clearing off-target infections. However, it was demonstrated that VSV replication, although slightly blunted, could be sustained inside tumors for up to 96 h post-administration, despite a pre-existing virus-specific T cell response^2^. Indeed, the replication of the virus over these 96 h was relatively robust as titers reached levels well above the input dose. Further, this phenomenon occurred despite the fact that vaccine-induced T cells can infiltrate tumors^10^. This created a conundrum because the tumor-specific T cells induced by our primary cancer vaccine could infiltrate tumors and were also specific for the transgene in VSV, which expressed the homologous antigen in infected cells; yet the VSV could replicate inside tumors. The research presented here directly addressed the question of how VSV could replicate inside a tumor microenvironment that harbored transgene-specific T cells. We hypothesized that two mechanisms could allow an OV to replicate in an environment pre-populated with transgene-specific T cells. First, it is commonly recognized that CD8^+^ cytotoxic T lymphocytes (CTLs) are an important antiviral effector subset^11^. However, tumor-infiltrating CTLs are often dysfunctional due to the numerous immunoevasion mechanisms employed by tumors^12,13^. Dysfunctional virus-specific T cells would have a reduced capacity to limit virus replication within a tumor. Secondly, viruses can cause acute lymphopenia in blood^14,15^. The mechanisms underlying virus-induced lymphopenia vary from death of lymphocytes^16,17^, to transient margination of lymphocytes in blood and/or lymphatic vessels due to acute virus-induced cytokine-mediated changes in expression of chemokine receptors and adhesion molecules^18–20^. We hypothesized that administering an OV would induce intratumoral lymphopenia prior to the completion of a virus replication cycle. To the best of our knowledge, this phenomenon had never been investigated before. If true, it would provide a novel mechanism by which virus-specific T cells could be cleared from a tumor, thereby facilitating VSV-mediated oncolysis. We focused on studying the potential for induction of intratumoral lymphopenia within 6 h of administering the oncolytic virus based on the rationale that this is less than the time required for a virus replication cycle, and, therefore, would be prior to the virus being able to mediate substantial presentation of antigens to local T cells. In these studies, secondary T cell responses were irrelevant since their expansion is not initiated until at least 48 h after administration of the VSV^3^. The presence of tumor-infiltrating CTLs with functional impairments at the time of administration of VSV, coupled with acute virus-induced intratumoral lymphopenia, could explain how intentional vaccination against a VSV-encoded transgene would enhance its safety profile while maintaining oncolytic activity.

## Results

### Inducing a primary immune response against a transgene encoded in a VSV-vectored booster vaccine made the OV safer

**Figure 1:**
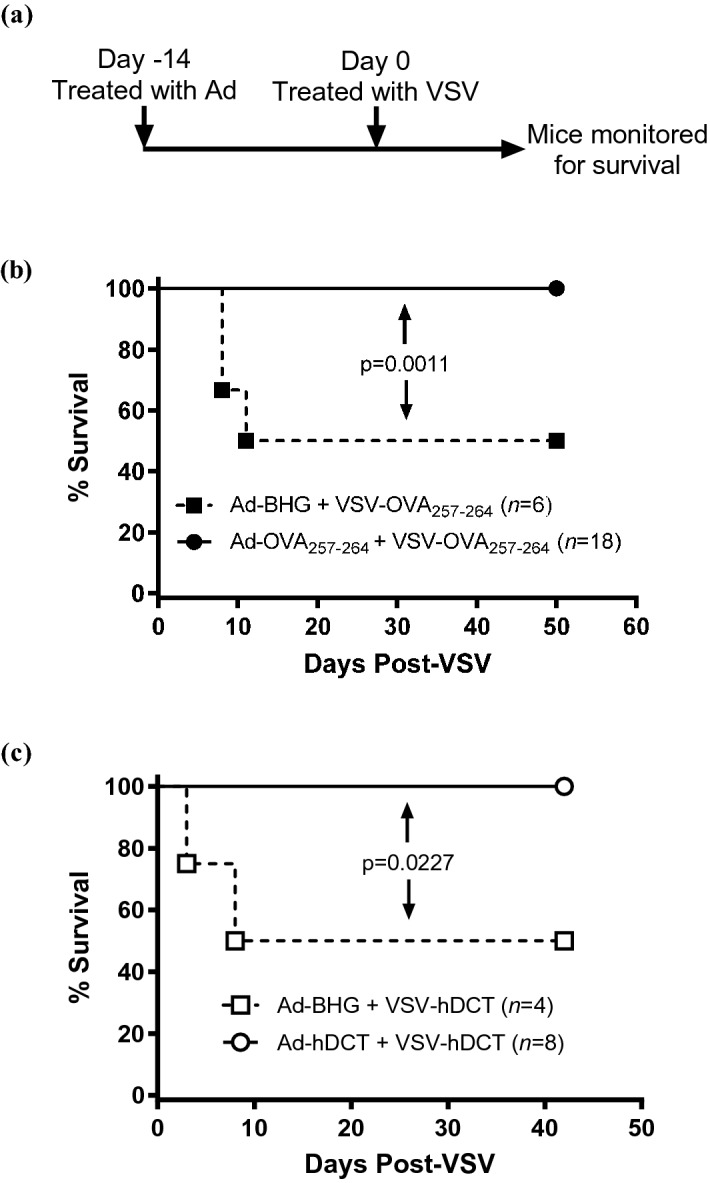
Inducing a primary immune response against an oncolytic virus-encoded transgene made the virus safer. **(a)** To assess the impact of primary vaccination on the safety of an oncolytic virus, when administered as a booster vaccine, tumor-free mice received intramuscular injections of 1 × 10^8^ IU of an E1/E3-deleted replication-deficient human serotype 5 adenovirus (Ad) with a transgene encoding an antigen on day −14. This was followed fourteen days later (on study day 0) by intravenous administration of 3 × 10^9^ pfu of vesicular stomatitis virus (VSV) encoding the same antigen. Survival was measured in days following treatment with VSV. Controls received an Ad that lacked a transgene but the same VSV-vectored vaccine as the test mice. Kaplan–Meier survival plots were calculated for **(b)** C57BL/6 or **(c)** Balb/c mice that were primed with Ad expressing **(b)** the immunodominant epitope from ovalbumin (OVA_257-264_) or **(c)** full-length human dopachrome tautomerase (hDCT) followed fourteen days later by intravenous boosting with VSV encoding the same antigen. Controls received adenoviruses that lacked a transgene (Ad-BHG). Mice were monitored and euthanized if they showed signs of reaching endpoint, which was always the onset of hind-limb weakness or paralysis. The data were analyzed using the Mantel-Cox log-rank method. Out of consideration for animal welfare, data shown in **(b,c)** were derived from single experiments.

Previous studies demonstrated that oncolytic rhabdoviruses expressing TAAs can dramatically boost tumor-specific T cell responses when used in heterologous prime-boost vaccine regimens^2–5,7,21,22^. In this setting, VSV was administered to hosts with a pre-existing transgene-specific T cell response. One would expect the safety profile of the VSV to be enhanced with its replication blunted by the T cells. Although we previously obtained retrospective safety data to support this^2^, no prospective studies had been done. Therefore, C57BL/6 and Balb/c mice were immunized with a replication-deficient adenovirus-vectored vaccine expressing OVA_257-264_ or DCT_180-188_, respectively. Controls were vaccinated with an adenovirus lacking a transgene. Mice were subsequently immunized with very high doses (3 × 10^9^ pfu, which was above the maximum tolerated dose of 1 × 10^9^ pfu) of VSV encoding the homologous transgene (Fig. 1). The parental recombinant strain of VSV (without the Δm51 mutation) was used for these toxicity studies since it is less attenuated than the Δm51 mutant and, therefore, potentially more toxic. Control mice were treated with an adenovirus lacking a transgene, followed by vaccination with VSV. All mice primed against the VSV-encoded transgene survived with no signs of toxicity. In contrast, 50% of mice that were immunologically naïve with respect to the VSV-encoded transgene, were euthanized due to onset of hind-limb paralysis, which is a common sign of VSV-induced toxicity due to neurotropism^9^. Both survival studies demonstrated complete abrogation of toxicity caused by VSV when it was administered to pre-vaccinated hosts (p ≤ 0.0227). Importantly, this was shown in two different strains of mice and for two different vaccine-encoded transgenes. This confirmed the obvious, that vaccinating against a transgene encoded by VSV makes the virus safer. However, this created a conundrum because a VSV booster vaccine encoding a homologous transgene was previously shown to replicate acutely inside tumors^2^. Therefore, we sought to uncover mechanisms that would allow this to occur.

### Boosting with VSV induced lymphopenia that rapidly removed transgene-specific T cells from tumors, thereby reducing their cytotoxic potential

**Figure 2:**
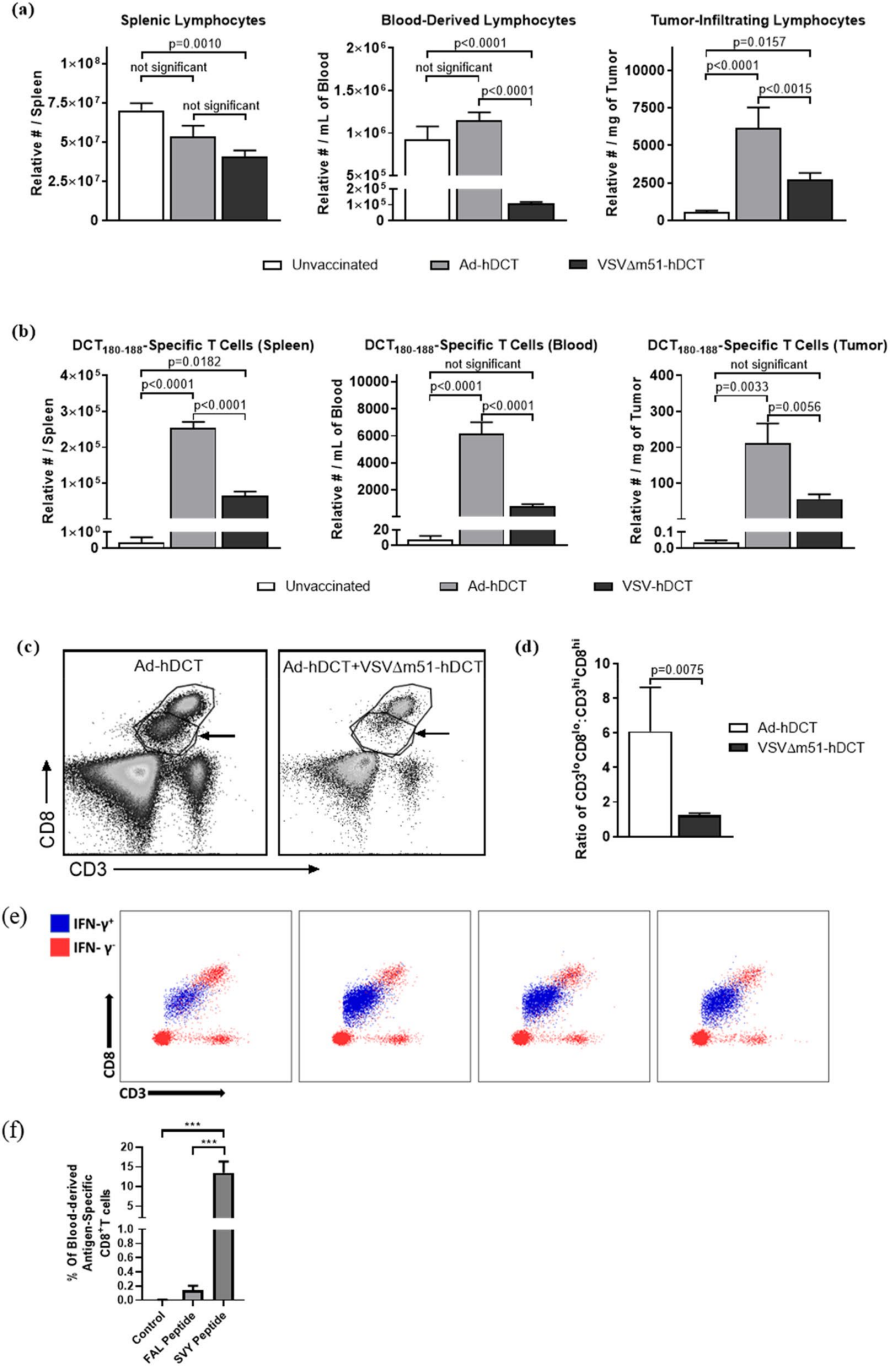
An oncolytic vesicular stomatitis virus-vectored booster vaccine induced acute lymphopenia, which was evident in the transgene-specific T cell subset. C57BL/6 mice with orthotopic B16-F10 melanomas (2.5 × 10^5^ cells injected intradermally) were unvaccinated, vaccinated intramuscularly with 1 × 10^8^ IU of an adenovirus encoding human dopachrome tautomerase (Ad-hDCT) or primed with Ad-hDCT four days post-challenge, followed ten days later by intravenous administration of 1 × 10^9^ pfu of vesicular stomatitis virus expressing hDCT (VSVΔm51-hDCT). Six- and twenty-four-hours post-VSV-hDCT, blood and saline-perfused spleens and tumors were harvested to quantify **(a)** lymphocytes (CD45^+^ cells in the lymphocyte gate shown in supplementary Fig. 1) by flow cytometry. **(b)** Similarly, hDCT-specific CD8^+^ T cells (defined as shown in supplementary Fig. 1) were quantified six hours post-VSV-hDCT. **(c)** The lymphopenic effect of oncolytic virotherapy was most prominent in the CD3^lo^CD8^lo^ blood-derived T cells (arrows), as demonstrated in the representative dot plots. **(d)** The ratios of CD3^lo^CD8^lo^:CD3^hi^CD8^hi^ T cells are shown. **(e)** Representative dot plots for four Ad-hDCT-vaccinated mice (10 days post-immunization) are shown after flow cytometric analysis following re-stimulation of blood-derived leukocytes with the DCT_180–188_ peptide. Interferon-gamma-producing T cells are overlaid in blue on the plots that show expression of CD3 and CD8. This is to demonstrate that activated antigen-specific T cells almost exclusively have a CD3^lo^CD8^lo^ phenotype. **(f)** The percentage of total CD8^+^ T cells that were specific for the immunodominant epitope from the adenoviral backbone (FAL) versus the melanoma-associated antigen (SVY) in blood. Controls were unvaccinated. These data were generated using an intracellular cytokine staining assay after re-stimulation with the peptides, followed by flow cytometric analysis. All graphs depict means and standard errors, with data pooled from three experiments (*n* = 16 unvaccinated control mice and *n* = 10 mice for both vaccinated groups). Results were analyzed by one-way analysis of variance with Tukey’s multiple comparison tests.

Curiously, VSV could transiently replicate inside the tumor of a host despite systemic distribution of pre-existing virus-specific T cells^2^. Lymphopenia is sometimes observed in blood in response to viral infections^14,23^. Although never shown before, we hypothesized that acute lymphopenia might also occur inside tumors of OV-treated hosts, resulting in a transient reduction in the number of CTLs capable of eliminating intratumoral viruses. To determine if lymphopenia occurred in response to VSV in vaccinated, tumor-bearing hosts, and whether this phenomenon extended to tissues other than blood, lymphocytes were quantified in blood, spleens and tumors by flow cytometry (Fig. 2a). C57BL/6 mice bearing orthotopic melanomas were unvaccinated, primed with Ad-hDCT, or primed with Ad-hDCT and boosted ten days later with VSVΔm51-hDCT. Tissues were harvested from all mice six hours after the one group was boosted. Assessment within such a short timeframe after boosting ensured that any changes in lymphocyte profiles were being defined prior to when substantial presentation of VSV-derived epitopes would be expected on infected cells. Indeed, it is generally accepted that that replication cycle of VSV is at least 6 h^24^ and additional time would then be required for transgene-derived antigen processing and presentation before an infected cell would potentially become a target for transgene-specific T cells. When compared to mice that received the primary vaccine only, spleen-derived lymphocytes were unaffected in the boosted group, but lymphopenia was evident in blood (Fig. 2a, left and middle graphs). Vaccination with Ad-hDCT induced higher numbers of TILs than unvaccinated controls but this was abrogated in mice boosted with VSV (Fig. 2a, right graph). Next, we assessed whether this lymphopenia extended to TILs specific for the virus-encoded transgene. Transgene-specific CTLs were quantified in the spleens, blood, and tumors of unvaccinated, Ad-hDCT-primed, and Ad-hDCT-primed and VSVΔm51-hDCT-boosted mice (Fig. 2b). As expected, DCT_180–188_-specific T cells were only present in substantial numbers in vaccinated mice. Notably, boosted mice had 75%, 87%, and 74% fewer antigen-specific CTLs in their spleens, blood, and tumors, respectively, as compared to the group that received the primary vaccine only. Activation of cytotoxic T cells can lead to downregulation of surface-expressed CD3 and CD8^25–27^. Analysis of the ratio of blood-derived CD3^lo^CD8^lo^:CD3^hi^CD8^hi^ cells suggested that those with the CD3^lo^CD8^lo^ phenotype were preferentially susceptible to OV-induced lymphopenia (Fig. 2c,d). In the murine model used in these studies the CD8^low^ cells were the functionally active tumor-associated antigen-specific T cells. Specifically, upon re-stimulation with the cognate peptide, the antigen-specific T cells reduced the concentration of CD8 on their surface (Fig. 2e).

In the studies presented here the adenovirus vector was replication-deficient but the transgene encoding the tumor antigen was under the control of a potent promoter. The strategy was to have transgene-encoded proteins dominate the protein milieu (i.e. the proteins from the viral vector backbone). This resulted in higher magnitude T cell responses against the transgene encoded by the adenovirus vector as compared to the immunodominant CD8^+^ T cell epitope from the vector backbone, even though the transgene was for a self epitope (i.e. the immunodominant CD8^+^ T cell epitope from dopachrome tautomerase). These results are shown in Fig. 2f and are a key reason why replication-deficient viral vectors have gained in popularity among vaccinologists.

**Figure 3:**
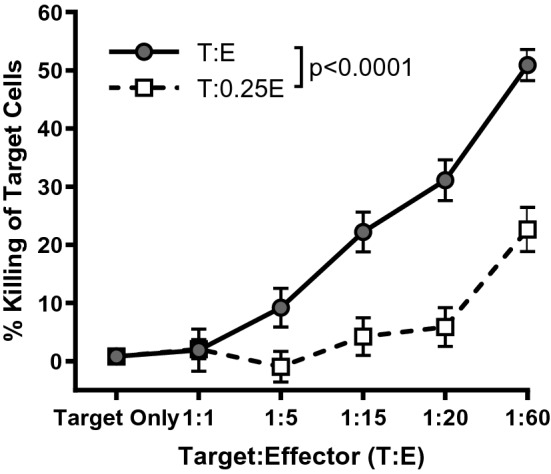
A four-fold reduction in the number of transgene-specific CD8^+^ T cells significantly abrogated cytotoxicity. C57BL/6 mice were vaccinated intramuscularly with 1 × 10^8^ IU of an adenovirus expressing human dopachrome tautomerase (hDCT). Ten days later, splenocytes were harvested and used for the negative magnetic selection of CD8^+^ T cells. These T cells were co-cultured with various ratios of B16-F10 melanoma cells that had been pre-treated for 24 h with recombinant interferon-gamma to upregulate the expression of major histocompatibility complex molecules to facilitate antigen presentation. In parallel, a second set of co-cultured cells were tested in which the number of effector cells were reduced four-fold. The cytotoxicity assay was run for 16 h. Means and standard errors are shown. The experiment was conducted once with eight replicates per group. Data were analyzed by two-way analysis of variance with Tukey’s multiple comparisons test.

To determine if the reduction of intratumoral transgene-specific CD8^+^ T cells shown in Fig. 2 was biologically relevant, an ex vivo cytotoxicity assay was performed using B16-F10 melanoma cells as targets (Fig. 3). A range of effector to target cell ratios was used. In parallel, a second set of effector to target ratios was tested in which the number of effector cells was reduced four-fold. Notably, a four-fold reduction in the number of effector T cells significantly abrogated the overall cytotoxicity (p < 0.0001). These results provided the first-ever evidence that VSV can transiently but rapidly remove most transgene-specific CTLs from a tumor microenvironment and this translated into a substantial reduction in their cytotoxic potential. However, this VSV-induced lymphopenia did not eliminate all the virus/transgene-specific TILs. Therefore, we explored a second possible mechanism that might support transient intratumoral replication of VSV in a host with pre-existing virus-specific immunity.

### TCRs of transgene-specific TILs were of lower functional avidity

**Figure 4:**
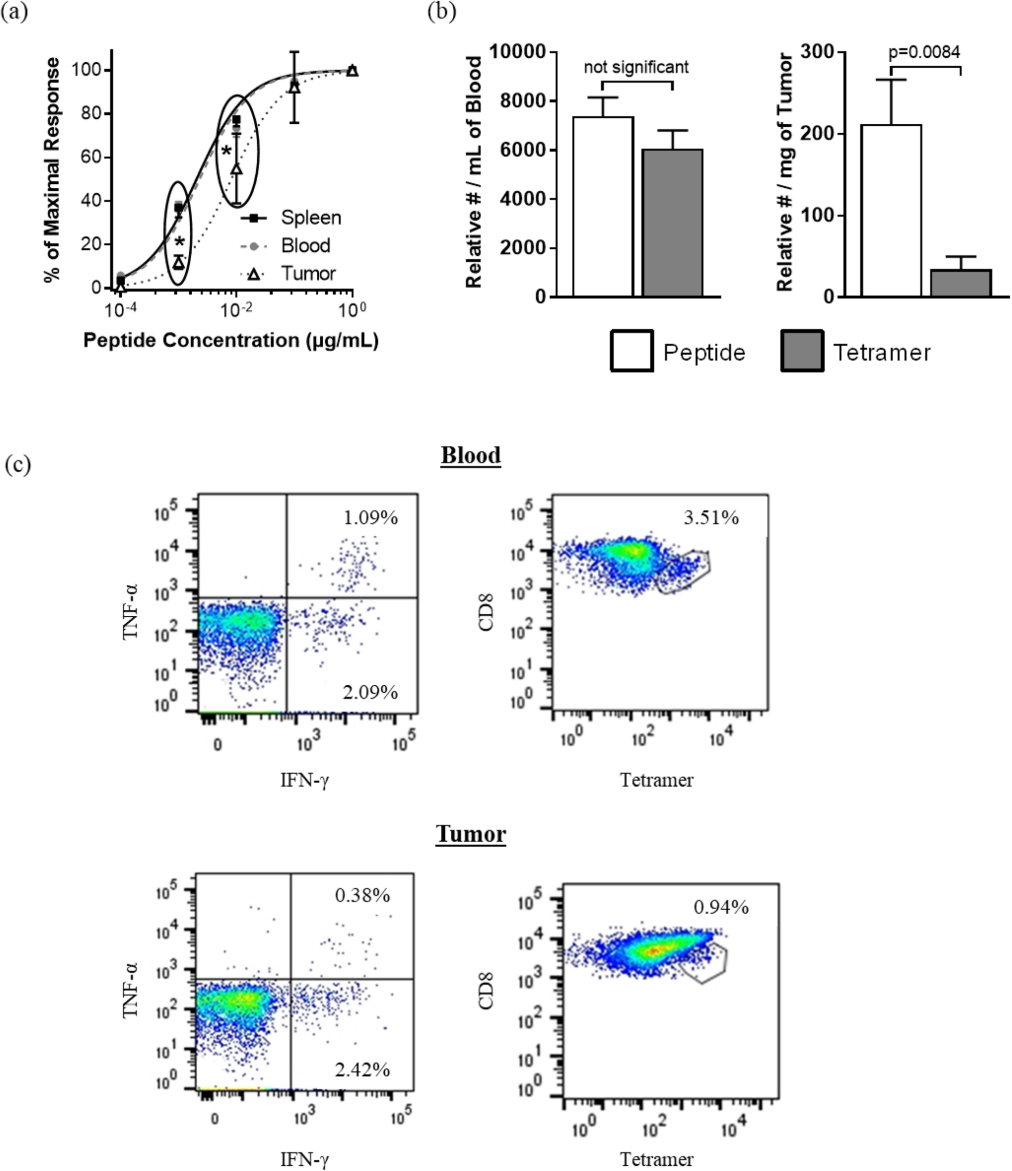
Transgene-specific tumor-infiltrating CD8^+^ T cells were of relatively low functional avidity. C57BL/6 mice received intradermal injections of 2.5 × 10^5^ B16-F10 melanoma cells. Four days later, they were vaccinated intramuscularly with 1 × 10^8^ IU of an adenovirus expressing human dopachrome tautomerase (hDCT). Ten days after immunization, blood and saline-perfused spleens and tumors were harvested for flow cytometric analysis of CD8^+^ T cells specific for the immunodominant epitope from DCT (DCT_180–188_). **(a)** The relative functional avidity of T cell receptors was compared between the three tissues through flow cytometric assessment of intracellular staining of IFN-γ after re-stimulating T cells with decreasing concentrations of DCT_180–188_. The frequencies of activated T cells were normalized to those exposed to the highest concentration of the cognate peptide (1 μg/mL). **(b)** To complement the functional avidity assay, T cells from the blood (left graph) and tumor-derived T cells (right graph) were quantified in two ways: (1) by intracellular cytokine staining of IFN-γ following re-stimulation with 1 μg/mL of the immunodominant epitope from DCT (DCT_180–188_), or (2) they were directly stained with DCT_180–188_-loaded tetramers. The scientific literature has shown that tetramer staining tends to be preferentially compromised by low concentrations of T cell receptors on lymphocytes as compared to the intracellular cytokine staining method and can, therefore, be used as an additional indirect assessment of functional avidity. **(c)** Representative dot plots from blood (upper graphs) and tumors (lower graphs) following intracellular cytokine staining (left graphs) or tetramer staining (right graphs). Data from three experiments were pooled (*n* = 12/group). Means and standard errors are shown. Results were assessed by **(a)** two-way analysis of variance with Tukey’s multiple comparisons test (*p < 0.05) or **(b)** unpaired two-tailed t-tests.

In addition to the novel finding that VSV could induce intratumoral lymphopenia, we suspected that CTLs would be rendered dysfunctional due to the immunosuppressive nature of tumors^12,13^. One potential defect is reduced functional avidity of CTLs due to a low concentration of surface-expressed T cell receptors and/or preferential recruitment of T cells with low-affinity receptors^28–30^. Therefore, we assessed the avidity of transgene-specific CTLs in Ad-hDCT-vaccinated mice with or without boosting with VSVΔm51-hDCT. Spleen-, blood-, and tumor-derived leukocytes were re-stimulated with serially log-diluted concentrations of the DCT_180–188_ peptide to generate tissue-specific dose–response curves. Fewer CTLs derived from tumors were able to respond to intermediate concentrations of the cognate peptide, as compared to spleen, and blood-derived CTLs (Fig. 4a). This was further confirmed by comparing numbers of blood-, and tumor-derived DCT_180-188_-specific T cells by intracellular cytokine staining after peptide re-stimulation versus direct flow cytometric detection of tetramer-stained cells. Compared to the intracellular cytokine staining method, tetramer staining tends to be compromised by low concentrations of cognate surface-expressed T cell receptors^27,31^. Indeed, the number of DCT_180-188_-specific T cells detected by tetramer staining was reduced in tumors but not blood, as compared to the number determined by intracellular cytokine staining (Fig. 4b). This suggested that tumor-infiltrating CTLs had relatively low functional avidity.

### Antigen-specific TILs were functionally impaired ex vivo and in vivo

**Figure 5:**
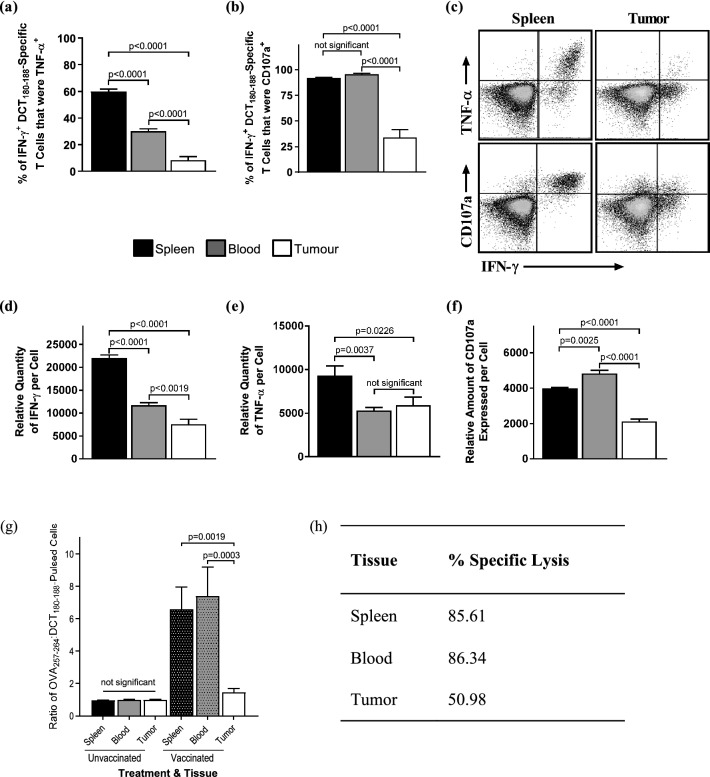
Transgene-specific CD8^+^ tumor-infiltrating T cells were functionally impaired ex vivo and inefficient at killing target cells in vivo. C57BL/6 mice with orthotopic B16-F10 melanomas (2.5 × 10^5^ cells injected intradermally) were vaccinated intramuscularly with 1 × 10^8^ IU of an adenovirus encoding human dopachrome tautomerase (Ad-hDCT) 4 days post-tumor challenge. Ten days after the immunization, blood, and saline-perfused spleens and tumors were harvested for ex vivo flow cytometric assessments of the functionality of CD8^+^ T cells specific for the immunodomiant epitope of DCT (DCT_180–188_) using an intracellular cytokine staining assay to detect production of IFN-γ and TNF-α. The proportion of T cells that were **(a)** multi-cytokine producers (i.e. making TNF-α in addition to IFN-γ) and **(b)** capable of degranulating (by quantifying antibody-mediated tethering of CD107a to the cell surface during re-stimulation) were analyzed. **(c)** Representative dot plots show the phenotypic differences between splenic and intratumoral DCT_180–188_-specific CD8^+^ T cells (i.e. those that are IFN-γ^+^) in terms of their ability to produce a second pro-inflammatory cytokine (i.e. TNF-α; upper plots) and degranulate (i.e. cells with CD107a tethered to their surface; lower plots). These plots also show the differences in the relative amounts (determined by mean fluorescence intensity) of IFN-γ, TNF-α, and surface-expressed CD107a on a per-cell basis, which were quantified in panels **(d–f)**, respectively. **(a–f)** Data were pooled from three experiments (*n* = 10 mice/group), with graphs showing means and standard error bars. Results were analyzed by one-way analysis of variance with Tukey’s multiple comparisons test. **(g)** The in vivo cytolytic potential of T cells was evaluated in spleens, blood, and tumors of intradermal B16-F10 melanoma-bearing C57BL/6 mice that were either unvaccinated (controls) or vaccinated intramuscularly with 1 × 10^8^ IU of an adenovirus vector expressing human dopachrome tautomerase (Ad-hDCT; test group). Ten days post-vaccination, mice received intravenous and intratumoral injections (doses split evenly between the two sites) of a 1:1 mixture of ovalbumin (OVA)_257–264_ and DCT_180–188_ peptide-pulsed splenocytes from naïve donors. Cells were labelled with different concentrations of a fluorescent dye to allow them to be distinguished. Tissues were harvested 13 h later to quantify the ratios of OVA_257–264_:DCT_180–188_-pulsed target cells by flow cytometry. Means and standard errors are shown, with data pooled from two experiments (*n* = 6 mice/group). Results were analyzed by one-way analysis of variance with Tukey’s multiple comparisons test. **(h)** The data from mice vaccinated with Ad-hDCT were summarized as mean percentage of target cell lysis in the three tissues that were assessed.

A reduction in T cell avidity is often associated with defects in cytokine production and degranulation^32–35^. Therefore, functions of DCT_180–188_-specific T cells from blood, spleens, and tumors were assessed ex vivo ten days after vaccination with Ad-hDCT (when the VSV-vectored booster vaccine would be administered). Qualitatively superior T cells can secrete multiple effector cytokines and efficiently degranulate^36–38^. In our model, antigen-specific T cells were identified as those that could produce IFN-γ in response to re-stimulation with their cognate peptide. To qualitatively assess DCT-specific cells, the percentage that could also produce TNF-α was determined. Further, the frequency of cells that underwent degranulation was identified by expression of CD107a on the surface of T cells during peptide re-stimulation. CD107a lines vesicles containing cytolytic granules and is transiently expressed during exocytosis^39^. This allows CD107a to be tethered to the cell surface if anti-CD107a is present during degranulation, facilitating detection by flow cytometry. Compared to splenic T cells, multi-cytokine-producing CTLs were reduced in frequency in the blood of melanoma-bearing mice (Fig. 5a,c). This defect was even more pronounced in tumor-infiltrating CTLs (p < 0.0001; Fig. 5a). There were also fewer tumor-infiltrating CTLs that could degranulate compared to CTLs in spleens and blood (Fig. 5b,c). Further, as compared to the spleen, the amount of IFN-γ that could be produced per CTL was decreased in blood and further decreased in tumors (Fig. 5d). TNF-α production by CTLs was also reduced on a per-cell basis in blood and tumors when compared to spleens (Fig. 5e). Finally, the relative degree to which CTLs could degranulate was actually better in blood when compared to spleens but decreased precipitously in tumors (Fig. 5f). Therefore, in addition to having reduced functional avidity, transgene-specific T cells inside tumors showed impaired cytokine production and degranulation. Motivated by ex vivo evidence of TIL dysfunction, an in vivo cytotoxicity assay was optimized to assess the ability of tumor-infiltrating CTLs to kill target cells in vivo. Syngeneic target splenocytes were pulsed with an irrelevant peptide (OVA_257–264_) or the immunodominant peptide from the vaccine-encoded antigen (DCT_180–188_), and differentially labelled with a fluorescent dye. Labeled cells were adoptively transferred into unvaccinated controls, or mice vaccinated with Ad-hDCT 10 days prior. The cytolytic ability of vaccine-induced CTLs was assessed in spleens, blood, and tumors thirteen hours post-transfer by determining ratios of recovered OVA_257–264_:DCT_180–188_-pulsed splenocytes by flow cytometry (Fig. 5g). As expected, this ratio was approximately 1:1 in all three tissues of unvaccinated mice, indicating a lack of DCT-specific cytolysis. In contrast, the OVA_257–264_:DCT_180–188_-pulsed splenocyte ratio was dramatically increased in the spleens and blood of vaccinated mice, suggesting there was DCT-specific killing. Importantly, this ratio was substantially lower for tumor-derived CTLs (p ≤ 0.0019); in fact, it did not differ significantly when compared to tumor-infiltrating CTLs from unvaccinated controls. We also determined the percentage of specific lysis of CTLs derived from the three tissues, which was reduced in tumors compared to blood and spleens (Fig. 5h). Overall results suggest that tumor-infiltrating CTLs were functionally impaired when VSV was being administered, thereby providing a second mechanism to explain why acute clearance of an OV within a tumor would be compromised despite pre-existing immunity against the OV-encoded transgene.

## Discussion

Cancer immunotherapy is an ideal approach to personalized medicine since it harnesses the inherent specificity and potency of a patient’s immune system to target neoplastic cells. Virus-mediated oncolysis is another modality that results in preferential killing of cancerous versus normal cells. In 2010 we published a strategy to synergize both approaches. Seminal research conducted by Richard Vile’s group and ours has shown the potential to use VSVs encoding TAAs as cancer vaccines^40–43^. Importantly, we demonstrated that VSV engineered to express a TAA could boost TAA-specific T cells to an unprecedented magnitude^2^, a strategy that was has been confirmed many times^3–5,7,21,22^ and is undergoing evaluation in human trials^8,44^. When this therapeutic strategy was published, a focus was placed on the transgene-specific T cells being tumor-specific. However, since these were directed against the transgene product encoded by the VSV booster vaccine, this created a problem that could not be explained until now: how could a VSV-vectored booster vaccine mediate direct oncolysis when the host has mounted a potent T cell response against the virus-encoded transgene? Indeed, the ability of a pre-existing transgene-specific immune response to protect against off-target toxicities mediated by high doses of VSV encoding the transgene has now been confirmed retrospectively^2^ and prospectively (Fig. 1). We reasoned that mechanisms to explain this problem might involve transient virus-induced lymphopenia leading to a decrease in the number of effector T cells capable of eliminating VSV-infected cancer cells, as well as tumor-mediated suppression of transgene-specific T cells remaining in the tumor. Note that VSV-vectored vaccines can induce high-magnitude transgene-specific antibody responses^21^. However, antibody responses are irrelevant to the therapeutic strategy being discussed here because the target antigen expressed by VSV is not incorporated into the virion.

Some viruses, including some OVs like VSV are known to induce profound, acute and transient lymphopenia^14,15^. Indeed, VSV-induced lymphopenia could occur within only three hours post-infection and reach a maximum loss of 90% of lymphocytes by twelve hours but return to pre-infection levels by 48 h^14^. This effect depended on type I interferons and could be recapitulated by administration of poly(I:C) or exogenous type I interferons instead of the virus^14^. Deletion of the type I interferon receptor from T and B cells confirmed that type I interferons act directly on lymphocytes to regulate recirculation, resulting in lymphopenia^45^. TNF-α can also promote lymphopenia^46^. These cytokines are potently induced by some OVs. Since VSV cannot efficiently infect resting lymphocytes, the phenomenon of VSV-induced lymphopenia has largely been attributed to a trafficking phenomenon rather than cytolysis. A key mechanism may be virus-induced cytokine-mediated upregulation of adhesion molecules on lymphocytes, causing them to stick to endothelial cells, a process known as margination^47^. Our results confirmed that VSV can induce a profound lymphopenia in blood by only six hours post-infection (Fig. 2). Although this was evident for both T and non-T lymphocytes, activated CD8^+^ T cells appeared to be preferentially affected by this phenomenon (Fig. 2c,d). We speculate that abnormal tumor vasculature lacking pericyte coverage might be impaired in its ability to express adhesion molecules like P- and E-selectin and intercellular adhesion molecule-1, which can support margination by binding L-selectin and leukocyte function-associated antigen-1 on lymphocytes^47^. If true, this would force tumor-infiltrating CTLs to marginate in normal blood vessels outside of the tumor during viral infection. Indeed, to our knowledge, these results provide the first evidence that intratumoral lymphopenia occurs rapidly following treatment with VSV (Fig. 2a), resulting in a loss of most of the CTLs specific for the virus-encoded transgene (Fig. 2b). This translated into a reduced capacity to kill cells expressing the target antigen (Fig. 3). This would temporarily reduce the capacity of a host to clear viruses from its own tumor. The rapid timing of this phenomenon would impair intratumoral presentation of transgene-encoded antigens, and likely those derived the viral backbone as well.

It is possible that the degree of intratumoral lymphopenia was underestimated in this study. Some of the blood vessels inside tumors likely retained at least partially normal architecture and should, therefore, have been able to support margination of lymphocytes. Also, purging all blood from tumors is not possible due to the torturous nature of the vasculature. Therefore, intratumoral transgene-specific T cells that were not in the tumor parenchyma may have been inadvertently quantified. Nevertheless, our data suggest that not all transgene-specific CTLs were removed from tumors, so we also evaluated the cytotoxic potential of TILs in our model.

Following vaccination, the number of TILs were increased (Fig. 2a), including transgene-specific CTLs (Fig. 2b). This was expected, since activated T cells tend to traffic into tumors^48^. However, we found that intratumoral T cells had numerous functional defects. These included reduced functional avidity (Fig. 4), a lower proportion of polyfunctional T cells (Fig. 4a,c), impaired degranulation (Fig. 5b,c), and a reduction in the cytokines produced per cell (Fig. 4d). Notably, the decreased cytokine production could be further exacerbated by the fact that tumor-infiltrating CTLs are reportedly impaired in their ability to release the comparatively few cytokines that they have^32^. These defects translated into a reduced ability of tumor-infiltrating CTLs to kill target cells expressing cognate epitopes in vivo (Fig. 5g). T cells are often functionally suppressed in tumors due to a variety of mechanisms, including the presence of regulatory T cells and myeloid-derived suppressor cells, indoleamine 2,3-dioxygenase-mediated catabolism of tryptophan that is required at high concentrations by CTLs, over-expression of immunological checkpoints, etc^49^. Tumor-induced immunosuppression is a well-described phenomenon that is usually associated with negative outcomes, like reduced immune-mediated killing of cancer cells^50^. This includes direct suppression of antigen-specific T cells^49^. However, in the context of an OV carrying a transgene being targeted by intratumoral CTLs, dampened TIL functions could be leveraged to facilitate early replication of the virus. This provides a practical demonstration of how the theoretical atavistic model, which is based on the well-described phenomenon of tumor-induced immunosuppression, can potentially be translated into clinical practice^51^.

Early efforts to improve therapies that relied on OVs focused on promoting their oncolytic potential. Largely based on observations that substantial anti-tumor efficacy could be obtained in immunodeficient but not immunocompetent mice^52^, the immune system was viewed as an enemy of oncolytic virotherapy^53^. In recent years, this attitude has changed with the realization that a more dominant mechanism of action mediated by OVs in immunocompetent hosts is their in situ vaccination effect. The therapeutic strategy that we developed, whereby an oncolytic rhabdovirus is used as a booster vaccine accepts that the immune system will clear the OV but we discovered that, in this context, direct oncolysis could still be achieved. The research presented here explains how this can happen. In summary, a VSV-vectored booster vaccine could rapidly and transiently deplete effector cells in tumors, thereby facilitating its own replication. Replication was likely further promoted by reduced functionality of the TILs that were present when administering the VSV. Admittedly, not all TILs were likely removed, nor were all dysfunctional, so VSV replication would be blunted to a degree and this is what was observed previously in two different cancer models spanning two strains of mice^2^. However, it did create a window in which VSV could replicate reasonably well. Our previous results suggested that this window was open for up to 96 h. By 72 h post-infection, secondary T cells started to proliferate^3^. Shortly thereafter, a massive secondary transgene-specific T cell response developed. If directed against a TAA, this response could kill cancer cells that survived initial virus-mediated debulking. Further, this secondary T cell response was of higher quality than the primary T cell repertoire, thereby helping to correct for a defect that helped support replication of the VSV in the first place^22^.

A secondary response to a VSV-encoded transgene could also clear off-target infections, thus making the OV safer than if administered to an unprimed host. The improved safety profile of a VSV-vectored booster vaccine was also promoted in the acute phase of replication by the fact that T cells in the spleen and blood remained more functional than their tumor-infiltrating counterparts, even though their numbers were also reduced due to virus-induced lymphopenia.

Proposing to prime a host against a VSV-encoded transgene to improve safety without abrogating oncolysis creates a new consideration for regulatory agencies. When testing a novel VSV in a clinical trial, regulators would typically require determination of the maximum tolerable dose of the virus as a monotherapy prior to considering combination therapies. However, this requirement would limit doses to levels that are below what could be tolerated in a primed host, thereby depriving patients of the optimal benefit of a VSV-vectored booster vaccine (Fig. 1). Therefore, we suggest that regulatory agencies consider allowing maximum tolerable doses of VSV-vectored secondary vaccines to be determined in combination with administration of the primary vaccine, not as stand-alone therapies.

Our novel finding of VSV-induced lymphopenia in melanomas might have additional implications. It begs the question of what acute effects OVs might have on trafficking of other leukocyte subsets. Recruitment of leukocytes into tumors is often described for oncolytic virotherapies^54^. However, our results demonstrated that VSV induced rapid and profound losses of lymphocytes from tumors, suggesting that the concept of OV-mediated increases in the number of TILs may need to be revisited for acute timepoints. It would also be important to determine if VSV-induced intratumoral lymphopenia extends to other OVs.

Importantly, the studies described here focused on acute mechanisms of action that facilitated transient replication of and, therefore, oncolysis mediated by VSV. This implies that the primary immune response was defective at the time of administration of the VSV-vectored booster vaccine. This prompts the need for clarification of the mechanism that contribute to the improved overall efficacy post-boost. Our previous publications have demonstrated that the over-riding advantages of boosting primary TAA-specific T cell responses with VSV include: 1. Direct oncolysis mediated by the oncolytic virus, 2. Boosting of the primary T cell response to massive numbers, and 3. enhancing the quality of the secondary T cells relative to their primary counterparts^2,3,22^.

One would not normally vaccinate against a virus for which replication in a host is desired. However, the mechanisms that we have demonstrated here provide a rationale for doing so. Having pre-existing virus-specific T cells provides a differential protective effect in tumors as compared to the rest of the body, due to transient VSV-induced intratumoral lymphopenia and the relative dysfunctional phenotype of tumor-infiltrating CTLs. These mechanisms allowed VSV to mediate direct, acute oncolytic effects, while enhancing its safety profile. This phenomenon of virus-induced intratumoral lymphopenia is novel and extends our understanding of host responses to viruses. Further, it introduces a new avenue of research in oncolytic virotherapy. Some scientists have started to question whether the field has moved too far towards the use of excessively attenuated viruses in the name of safety but at a cost of reduced efficacy. Being able to pre-vaccinate against lymphopenia-inducing VSVs raises the possibility of reversing this paradigm; less attenuated viruses with enhanced oncolytic potential could potentially be used without compromising safety. However, caution should be taken before trying to advance less attenuated VSVs to the clinic since many cancer patients have compromised immune systems. Recommendations to support translation of less attenuated viruses to clinical scenarios include first extending the findings reported here to other tumors, other lymphopenia-inducing OVs and other animal models, including humanized mice, to reliably prove safety. Further, pre-existing T cell responses against VSV-encoded antigens in cancer patients should not preclude the use of oncolytic virotherapy and may even make it safer. Finally, intentional vaccinations against OVs could, in theory, target any virus-encoded transgene. In the context of cancers such as glioblastomas, for which target antigens are poorly defined, pre-vaccinating against a virus encoded transgene that is not a TAA (e.g. a transgene to facilitate imaging or some other therapeutic transgene) or an antigen derived from the viral backbone could take advantage of this strategy. However, we would argue that a major advantage of making the target a TAA is that the virus-specific T cells that ultimately clear infections in the post-lymphopenia phase would also kill uninfected cancer cells. Whether the principles presented here extend to other OVs remains to be determined.

## Materials and methods

### Mice

Female C57BL/6 and Balb/c mice (Charles River Laboratories, Wilmington, Massachusetts, USA; strain #027 and #028, respectively) were 8–10 weeks old when experiments began and were housed in a specific pathogen-free, environmentally-controlled biological safety level-2 isolation facility at the University of Guelph (Guelph, Ontario, Canada). Food and water were provided ad libitum and mice were accommodated to their environment for one week before experimentation. Mouse studies were approved by the University of Guelph’s Animal Care Committee (animal utilization protocol #3807) and complied with the relevant guidelines and protocols published by the Canadian Council on Animal Care. The mouse studies were also carried out in compliance with the ‘Animal Research: Reporting of In Vivo Experiments’ guidelines^55^.

### Cell cultures

HEK-293, Vero and murine B16-F10 melanoma cells [American Culture Collection^®^ (ATCC), Manassas, Virginia, USA; lines #CRL-1573, #CCL-81 and #CRL-6475, respectively] were cultured in HyClone^®^ Dulbecco’s Modified Eagle Medium [Fisher Scientific, Ottawa, Ontario, Canada; catalogue (cat.) #SH3002201] supplemented with 10% HyClone™ Bovine Calf Serum, U.S. Origin (Fisher Scientific Canada; cat. #SH3007203).

### Viruses

A recombinant E1/E3-deleted replication-deficient human serotype-5 adenovirus (Ad-BHG; Agilent Technologies, Mississauga, Ontario, Canada; cat. #240009) was previously engineered with a transgene encoding the full-length melanoma-associated antigen human dopachome tautomerase (Ad-hDCT)^40,56^. Ad-hDCT was propagated in HEK-293 cells, purified by ultracentrifugation on a cesium chloride gradient and titered by counting hexon-positive cells in confluent monolayers of HEK-293 cells using light microscopy after staining with a hexon-specific antibody (clone 8C4; Abcam, Toronto, Ontario, Canada; cat. #ab8249). For safety studies, two recombinant Indiana serotype VSVs were used. One expressed full-length hDCT (VSV-hDCT) and was described previously^40^. Similarly, a VSV expressing the immunodominant CD8^+^ epitope (for C57BL/6 mice) from chicken ovalbumin (OVA_257–264_, peptide sequence: SIINFEKL) was constructed (VSV-OVA_257–264_). All other vaccine studies used VSV that had the methionine at position 51 of the matrix protein deleted and was engineered to express hDCT (VSVΔm51-hDCT). The Δm51 mutation is attenuating by abrogating the type I interferon-blocking potential of VSV. VSV vector systems were provided by Brian Lichty, McMaster University, Hamilton, Ontario, Canada. Propagation and titration (by plaque assays) of VSVs were performed with Vero cells, with concentration and purification done as previously described^40^.

### Orthotopic melanoma model

C57BL/6 mice received intradermal injections on the back, between the scapulae, of 2.5 × 10^5^ B16F10 cells in 30 µL of phosphate-buffered saline (PBS).

### Vaccination

Anesthetized mice were immunized with 1 × 10^8^ infectious units (IU) of Ad-hDCT in 100 µL of PBS (50 µL/hamstring) and intravenous (i.v.) injection of 1 × 10^9^ plaque-forming units (pfu) of VSV in 200 µL of PBS.

### Antibodies and tetramer for flow cytometry

Flow cytometry assays used the following monoclonal antibodies: anti-CD16/CD32 (clone 93; Fisher Scientific; cat. #14-016186) to block Fc receptors, anti-CD3-brilliant violet-421 (clone 145-2C11; BD Biosciences; cat. #562600), anti-CD8-brilliant violet-510 (clone 53–6.7; BD Biosciences; cat. #563068) and anti-CD4-FITC (clone RM4-4; Fisher Scientific; cat. #11-0043-85) for detecting T cells, Fixable Viability Dye-eFluor780 (Fisher Scientific; cat. #65-0865-14) to exclude dead cells, and anti-IFNγ-APC (clone XMG1.2; Fisher Scientific; cat. #17-7311-82) and anti-TNFα-PE (clone MP6-XT22; Fisher Scientific; cat. #12-7321-82) for intracellular cytokine staining, and anti-CD107a-PE-Cy7 (clone eBio1D4B; Fisher Scientific; cat. #46-1071-82) for detecting degranulation during peptide-mediated re-stimulation of T cells. Violet Proliferation Dye (VPD)-450 (BD Biosciences; cat. #562158) was used to label donor splenocytes for in vivo cytotoxicity assays. DCT_180–188_-specific T cells were directly labeled with a H-2 Kb/DCT_180–188_-PE tetramer (Baylor College of Medicine, Houston, Texas, USA). Samples were analyzed with a FACS Canto II with FACSDiva 8.0.1 software (BD Biosciences), with data analyzed using FlowJo version 10.1 software (FlowJo LLC, Ashland, Oregon, USA).

### Analysis of T cell responses

Blood collected from the periorbital sinus of mice had erythrocytes lysed. Excised spleens were pressed between frosted ends of sterile glass microscope slides to make single-cell suspensions, followed by lysis of erythrocytes. For assessing TILs, mice were transcardially perfused with PBS to purge blood, then intradermal tumors were excised, weighed, minced, and dissociated with a gentleMACS™, according to the manufacturer’s instructions (Miltenyi Biotec, San Diego, California, USA). Dissociated TILs were filtered through a 70 µm strainer. Blood-, spleen, and tumor-derived cells were re-stimulated with 1 µg/mL of the DCT_180–188_ peptide (amino acid sequence: SVYFFVWL; shared by human and murine DCT; Pepscan, Lelystad, Netherlands) or the immunodominant CD8^+^ T cell epitope from the adenoviral vector backbone (amino acid sequence: FALSNAEDL; DNA-binding protein_418–426_^57^; Pepscan) in the presence of brefeldin A (1 µg/mL; added after 1 h of incubation; Fisher Scientific; cat. #501129757). After five hours of re-stimulation, cells were treated with anti-CD16/CD32 to block Fc receptors and phenotyped with fluorescently-labelled surface-staining antibodies. Cells were permeabilized and fixed (Intracellular Fixation Buffer and Perm/Wash Buffer, Fisher Scientific; cat. #00-8333 and #BDB554723, respectively) and stained for intracellular cytokines.

### Ex vivo cytotoxicity assay

C57BL/6 mice were vaccinated intramuscularly with 1 × 10^8^ IU of Ad-hDCT. Ten days later, splenocytes were harvested and used for the negative magnetic selection of CD8^+^ T cells (EasySep™ Mouse CD8 + T Cell Isolation Kit, StemCell Technologies, Vancouver, British Columbia, Canada, cat. #19853). These T cells were co-cultured with various ratios of B16-F10 melanoma cells that had been pre-treated for 24 h with recombinant interferon-gamma (BioLegend, cat. #575308) to upregulate the expression of major histocompatibility complex molecules to facilitate antigen presentation^58^. In parallel, a second set of co-cultured cells were tested in which the number of effector cells were reduced fourfold. The flow cytometry-based cytotoxicity assay was conducted as described previously^59^.

### Avidity assay

The intracellular cytokine staining assay was used with blood-, spleen-, and tumor-derived leukocytes that were stimulated with serially log-diluted concentrations of the DCT_180-188_ peptide.

### In vivo cytotoxicity assay

After lysis of erythrocytes, donor splenocytes were seeded into uncoated 60 mm cell culture plates and incubated for four hours. Non-adherent cells (*i.e*. enriched for lymphocytes) were collected and pulsed with the OVA_257–264_ (Pepscan) or DCT_180-188_ peptides at 10 µg/mL for 1 h at 37 °C and then stained with 0.2 and 1 µM of VPD-450, respectively. OVA_257–264_- and DCT_180–188_-pulsed cells were combined at equal numbers. Preliminary experiments demonstrated that trafficking of target cells into tumors was inefficient if they were only administered i.v. Therefore, half of the peptide-pulsed donor cells were administered intratumorally in 100 µL of PBS, and the other half were injected i.v. in 200 µL of PBS. Tumors, blood, and spleens were harvested 13 h later and viable, VPD-450-labeled cells were quantified by flow cytometry. Percentages of specific lysis were calculated for each tissue using the formula:

$$100{ } - { }\left[ {\frac{{\left( {\frac{VPDhigh}{{VPDlow}}} \right)vaccinated}}{{\left( {\frac{VPDhigh}{{VPDlow}}} \right)unvaccinated \, average}}} \right]{ } \times 100.$$

### Statistics

GraphPad Prism 7 for Windows (GraphPad software, San Diego, California, USA) was used for graphing and statistical analyses. Survival curves were assessed by the Kaplan–Meier method, with differences between groups queried using the log-rank test. Differences in mean cell numbers and frequencies were analyzed by unpaired t-tests (for two treatment groups) or one-way analysis of variance (ANOVA) with Tukey’s multiple comparisons test (when comparing > 2 treatments). In vivo cytotoxicity ratios were analyzed by one-way ANOVA. Graphs (except survival data) show means and standard errors. Differences between means were considered significant at p ≤ 0.05.
